# Spectral computed tomography as a novel diagnostic tool for mushroom poisoning–induced myocarditis: a case report

**DOI:** 10.1093/ehjcr/ytag316

**Published:** 2026-05-04

**Authors:** Haiyan Yang, Jinqiu Ruan, Saiyi Chen, Mei Su

**Affiliations:** Department of Radiology, The People’s Hospital of Chuxiong Yi Autonomous Prefecture, Chuxiong, Yunnan 675000, China; Department of Radiology, The People’s Hospital of Chuxiong Yi Autonomous Prefecture, Chuxiong, Yunnan 675000, China; Department of Radiology, The People’s Hospital of Chuxiong Yi Autonomous Prefecture, Chuxiong, Yunnan 675000, China; Department of Radiology, The People’s Hospital of Chuxiong Yi Autonomous Prefecture, Chuxiong, Yunnan 675000, China

**Keywords:** Toxic myocarditis, Spectral computed tomography, Extracellular volume, Myocarditis, Case report

## Abstract

**Background:**

Spectral computed tomography (CT) offers a promising ‘one-stop’ assessment for acute myocarditis, yet its diagnostic value in rare toxic aetiologies, such as mushroom poisoning, remains underexplored.

**Case summary:**

A previously healthy 58-year-old man presented with acute heart failure and multiorgan dysfunction following *Trogia venenata* mushroom ingestion. Initial troponin was mildly elevated. Emergency spectral CT coronary angiography excluded obstructive disease. Crucially, delayed-phase quantitative mapping revealed a globally increased myocardial extracellular volume (ECV: 33%–40%), indicating diffuse interstitial injury despite unremarkable conventional iodine maps. This finding was subsequently validated by cardiac magnetic resonance (CMR), which also showed globally elevated native T1 and ECV without late gadolinium enhancement, confirming the diagnosis of diffuse interstitial myocarditis.

**Discussion:**

This case underscores a pivotal role for spectral CT in diagnosing toxic myocarditis. It provides a rapid, comprehensive alternative when CMR is not readily available, uniquely quantifying diffuse injury via ECV mapping—a key biomarker that may be inconspicuous on conventional imaging. This ‘one-stop’ approach can expedite critical management decisions.

Learning points

- Maintain a high index of suspicion for toxic myocarditis in mushroom-ingestion patients presenting with acute heart failure, after acute coronary syndrome has been excluded.
- Spectral computed tomography is a crucial alternative when cardiac magnetic resonance is not feasible, as its extracellular volume mapping aids in diagnosing toxic myocarditis by quantifying diffuse interstitial injury.

## Introduction

Mushroom poisoning is a global health concern, with cardiac complications such as acute myocarditis being particularly rare and diagnostically challenging.^1,2^ The clinical presentation of acute myocarditis is highly variable and often mimics acute coronary syndrome (ACS), necessitating both the exclusion of coronary disease and objective evidence of myocardial injury for diagnosis.^3,4^ While cardiac magnetic resonance (CMR) is the established non-invasive reference standard for characterizing myocardial inflammation, its utility can be limited in emergency settings due to availability, long scan times, or patient instability.^4,5^ Spectral computed tomography (CT) has emerged as a promising alternative, offering a ‘one-stop’ assessment. It enables comprehensive coronary computed tomography angiography (CCTA) to rule out ACS while concurrently providing functional tissue characterization through late iodine enhancement (LIE) and quantitative extracellular volume (ECV) fraction mapping.^4,5^ This case report describes a patient with fulminant myocarditis following ingestion of the poisonous mushroom *Trogia venenata* and highlights the distinctive diagnostic value of spectral CT in this critical clinical scenario.

## Summary figure

**Figure:**
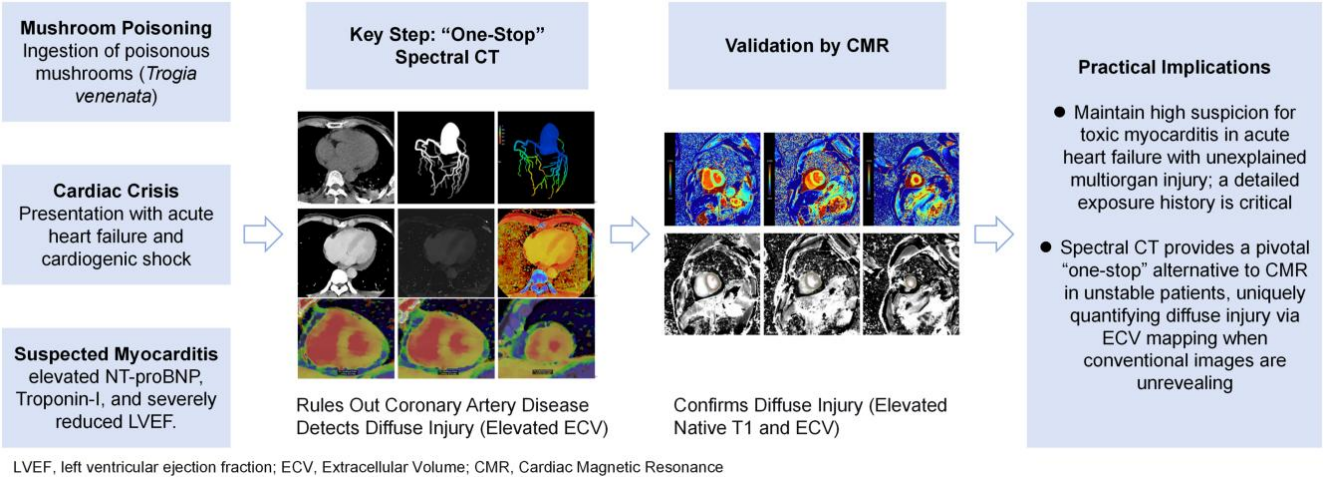


## Case presentation

We present a 58-year-old previously healthy man who developed fulminant myocarditis after ingesting *T. venenata*, a mushroom linked to clusters of sudden unexplained death as documented in the cited literature.^6,7^ Following self-limiting gastrointestinal symptoms, he presented to the emergency department with limb numbness, weakness, and signs of acute heart failure including tachycardia (114 b.p.m.), hypotension (106/71 mmHg), and hypoxaemia (PaO_2_ 77 mmHg). Electrocardiography revealed sinus tachycardia, incomplete right bundle branch block, right axis deviation, and non-specific ST-T changes with T-wave flattening and inversion (see Supplementary material online, *Figure S1*).

Laboratory tests confirmed severe myocardial stress and multiorgan dysfunction: markedly elevated N-terminal pro-B-type natriuretic peptide (NT-proBNP: 19 600 pg/mL) and C-reactive protein (12.36 mg/L), with mildly elevated troponin I (0.0248 ng/mL). Concurrent hepatic and renal injury was evident. Echocardiography showed severely reduced left ventricular ejection fraction (28%) with diffuse hypokinesis (see Supplementary material online, *Figure S2*). A diagnosis of cardiogenic shock was established based on sustained hypotension and signs of end-organ hypoperfusion.

**Figure 1:**
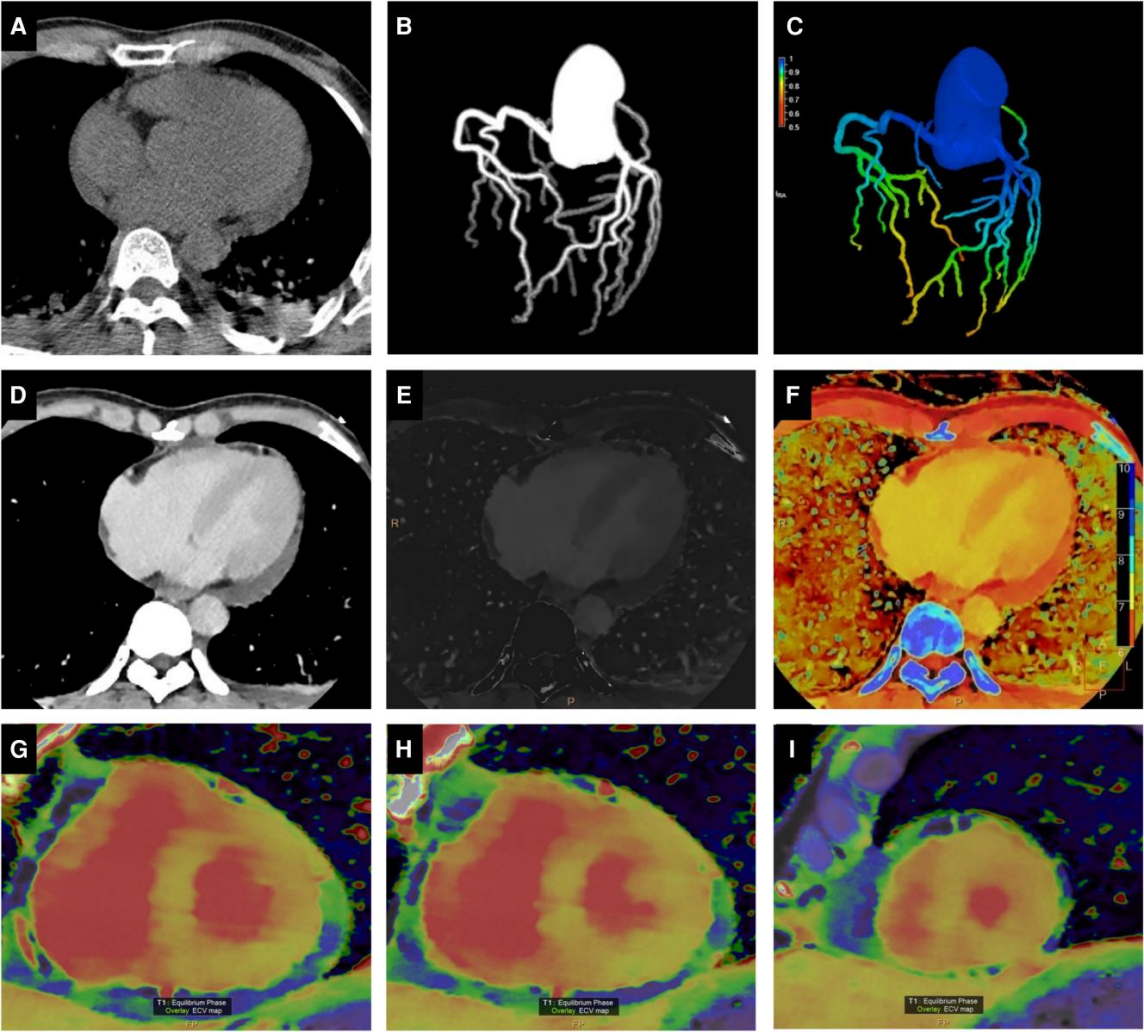
Comprehensive ‘one-stop’ cardiac assessment with spectral CT. (*A–C*) Anatomical and haemodynamic evaluation of coronary arteries. (*A*) Ungated non-contrast computed tomography scan showing no coronary artery calcification. A trivial pericardial effusion is observed. (*B*) Volume rendering reconstruction of the coronary tree demonstrates patent left anterior descending, left circumflex, and right coronary artery without significant stenosis. (*C*) CT-derived fractional flow reserve assessment. The colour-coded map overlaid on the coronary model shows FFR values > 0.80 (non-ischaemic) throughout all major epicardial vessels. (*D–F*) Myocardial tissue characterization on delayed-phase (7-min) spectral computed tomography. (*D*) Monoenergetic image at 40 keV. (*E*) Iodine density map. (*F*) Z-eff map. Visual assessment of panels (*D*)–(*F*) revealed no visually distinct focal areas of abnormal attenuation, iodine concentration, or Z-eff values in the left ventricular myocardium. (*G–I*) Quantitative extracellular volume mapping. Quantitative colour-coded extracellular volume maps at the (*G*) basal, (*H*) mid, and (*I*) apical ventricular levels demonstrate globally elevated extracellular volume values (ranging from 33% to 40%), consistent with diffuse interstitial expansion. The normal reference range for myocardial extracellular volume is typically 25% ± 3%.

**Figure 2:**
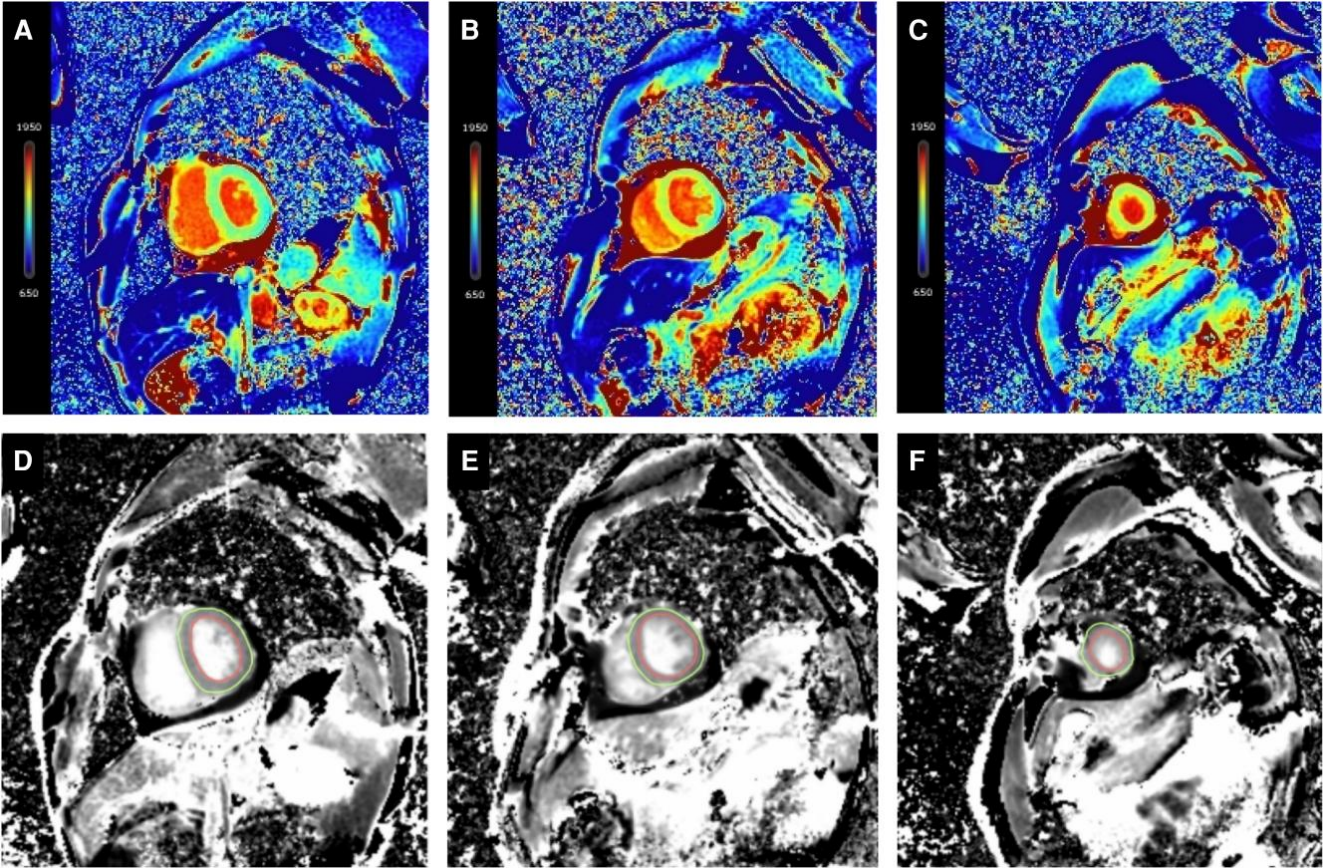
Cardiac magnetic resonance validation of diffuse myocardial injury. (*A–C*) Native T1 mapping. Native T1 maps at the (*A*) basal, (*B*) mid, and (*C*) apical ventricular levels show diffusely elevated T1 values throughout the left ventricular myocardium, indicative of myocardial oedema and/or interstitial expansion. (*D–F*) Extracellular volume mapping. Corresponding extracellular volume maps at the (*D*) basal, (*E*) mid, and (*F*) apical levels confirm globally increased extracellular volume, quantitatively confirming the diffuse interstitial pathology.

Emergency spectral CT was performed to rule out ACS using a dual-layer spectral CT scanner (Spectral CT Plus, Philips Healthcare). Coronary computed tomography angiography and CT-derived fractional flow reserve (CT-FFR) excluded obstructive disease. A delayed-phase spectral CT scan was acquired 7 min post-contrast injection of 60 mL iohexol (350 mgI/mL) administered at a flow rate of 5.0 mL/s. Haematocrit (40.6%) obtained on the same day was used for ECV calculation. Although monoenergetic (40 keV) images, iodine density maps, and Z-eff maps showed no visually apparent focal abnormalities, ECV mapping revealed globally elevated values (33%–40%). These values exceed the previously reported diagnostic threshold for acute myocarditis (31.60%) using CT-derived ECV,^8^ supporting the diagnosis of diffuse myocardial injury (*Figure 1*). Subsequent CMR confirmed globally elevated native T1 and ECV without late gadolinium enhancement, consistent with diffuse interstitial myocarditis (*Figure 2*).

The patient received mechanical circulatory support with an intra-aortic balloon pump maintained for 4 days, alongside non-invasive ventilation, diuresis for pulmonary oedema, and comprehensive therapy for myocardial, metabolic, and liver support, etc. A multidisciplinary approach was maintained to support end-organ function and promote recovery. He responded favourably and was eventually discharged in improved condition.

At 3-month telephone follow-up, the patient remained asymptomatic and had resumed normal daily activities without any limitations (New York Heart Association functional class I). No recurrent arrhythmias or heart failure symptoms were reported.

## Discussion

This case underscores the potential diagnostic utility of spectral CT in the acute evaluation of toxic myocarditis. Its principal advantage lies in its comprehensive, ‘one-stop’ assessment capability. It reliably excluded acute coronary occlusion via CCTA and CT-FFR, which is a crucial first step in the diagnostic algorithm for myocarditis.^4,5^ Beyond anatomy, spectral CT provided unique quantitative tissue characterization. Although visual assessment of conventional iodine maps showed no focal abnormalities, the globally and significantly elevated myocardial ECV (33%–40%) served as an objective, quantitative biomarker of diffuse interstitial oedema and injury. This finding correlated with the CMR results of elevated native T1 and ECV in the absence of late gadolinium enhancement (LGE), collectively indicating a myocardial injury pattern dominated by diffuse interstitial inflammation rather than confluent necrosis. Unlike conventional CT, spectral CT allows direct iodine quantification via material decomposition, ensuring more reliable ECV measurement. In emergency or critical care settings, spectral CT may shorten diagnostic time, reduce patient transport risks, and lower contrast exposure compared to traditional pathways.

Several differential diagnoses were considered and excluded. Takotsubo cardiomyopathy, which can simulate both ACS and acute myocarditis and is often clinically difficult to distinguish, was considered unlikely due to the absence of apical ballooning and reversible wall motion abnormalities on echocardiography. Moreover, CMR findings did not show the typical Takotsubo pattern—such as focal myocardial oedema with patchy LGE distribution.^9^ Myocardial infarction with non-obstructive coronary arteries was excluded by the absence of coronary stenosis on CCTA/CT-FFR and the lack of subendocardial or transmural LGE on CMR. Septic cardiomyopathy was considered unlikely given no clinical evidence of sepsis (normal white blood cell count, no fever). The combination of diffuse interstitial injury on imaging, temporal relationship to mushroom ingestion, and multiorgan dysfunction strongly supported toxic myocarditis.

Studies have shown high concordance between spectral CT LIE and CMR LGE for detecting focal inflammatory lesions (sensitivity 100%, accuracy 95%).^5^ Our case further demonstrates that even in diffuse patterns without typical LIE/LGE findings, spectral CT's quantitative ECV mapping can sensitively detect subtle myocardial changes. This is particularly significant when CMR is not immediately feasible due to haemodynamic instability, contraindications, or logistical constraints, allowing for prompt diagnostic triage and treatment initiation.^4^ In this case, the diffuse rather than focal pattern of injury meant that conventional contrast-enhanced imaging might miss the diagnosis, whereas quantitative ECV mapping served as the key diagnostic basis. A limitation of this report is the absence of endomyocardial biopsy confirmation, a common challenge in clinical myocarditis diagnosis.^3^ Future studies are warranted to define diagnostic thresholds for spectral CT parameters (e.g. ECV, iodine concentration) across various myocarditis aetiologies and to explore their prognostic value.

While previous case reports documented mushroom-induced myocarditis diagnosed by CMR,^3^ our case demonstrates the value of spectral CT in the acute setting where CMR was not feasible. This represents a novel application of spectral CT in the diagnostic workup of suspected toxic myocarditis from rare mushroom poisoning, highlighting its potential in this challenging clinical context.

In conclusion, this case highlights the pivotal role of multiparametric spectral CT as a rapid, comprehensive, and ‘one-stop’ imaging tool in the diagnostic workup of fulminant toxic myocarditis. It not only reliably excludes ACSs but also uniquely quantifies diffuse interstitial myocardial injury through ECV mapping, offering a critical diagnostic alternative when CMR is not feasible. This approach holds significant promise for improving the timely diagnosis and management of similar critical cardiac toxicities.

## Supplementary Material

ytag316_Supplementary_Data

## Data Availability

Non-identifiable data underlying this article will be made available upon reasonable request to the corresponding author.

## Lead author biography

Haiyan Yang, M.B., is a diagnostic radiologist at the Department of Radiology, The People’s Hospital of Chuxiong Yi Autonomous Prefecture. She focuses on emergency and cardiovascular diagnosis, applying multimodal cardiac imaging including CT and CMR to improve risk stratification.

## Notes

**Consent:** We have received the patient’s informed consent for the use of data from their medical records and clinical images obtained for publication, in accordance with COPE guidelines.
