# Supplementary material for: Spectral computed tomography as a novel diagnostic tool for mushroom poisoning–induced myocarditis: a case report
Source: Eur Heart J Case Rep. 2026 May 4;10(5):ytag316. doi: 10.1093/ehjcr/ytag316 (PMC13197056; doi:10.1093/ehjcr/ytag316)
Supplement: ytag316_Supplementary_Data [file ytag316_supplementary_data.docx]

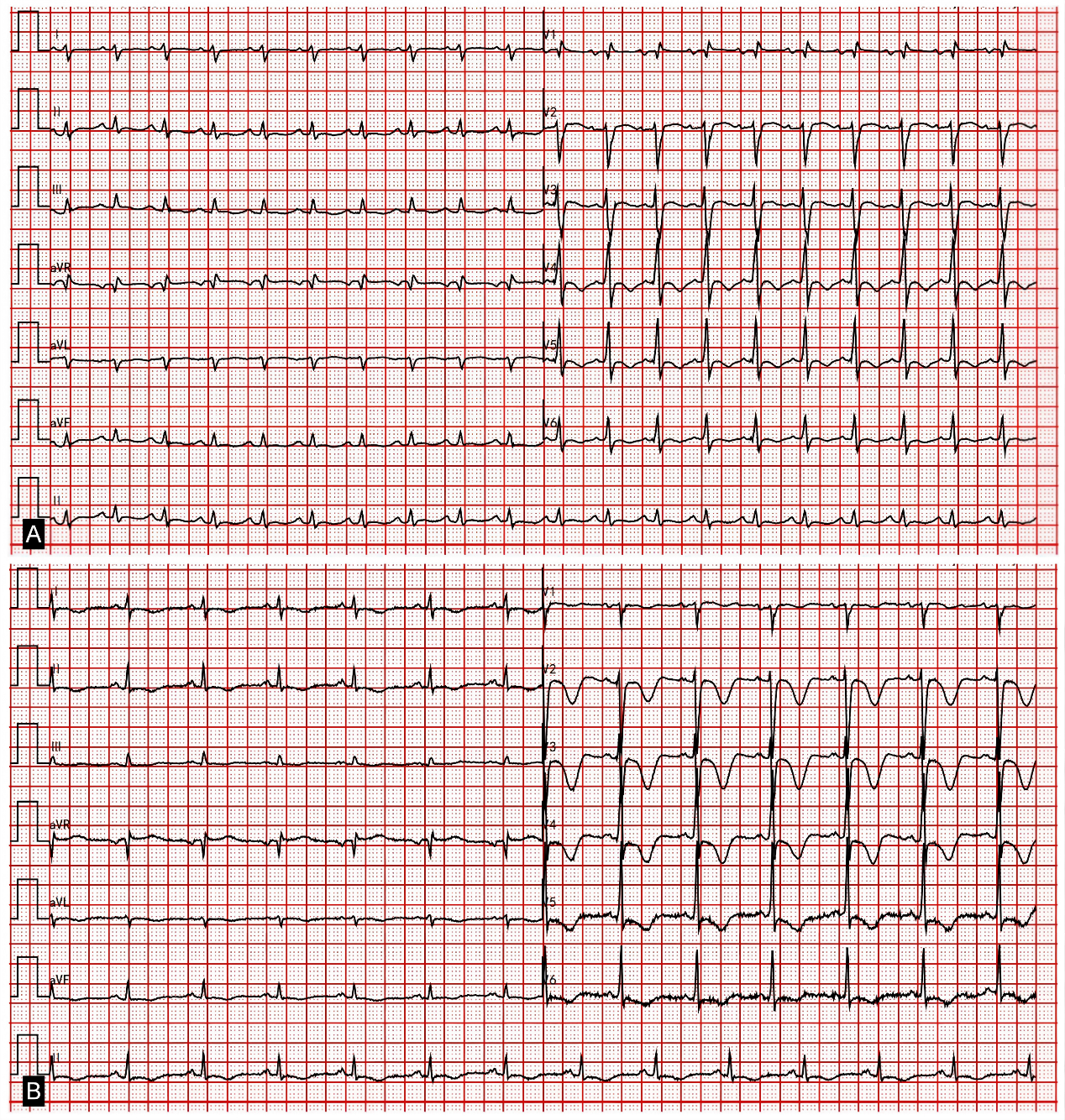


**Supplementary Figure S1. Twelve-lead electrocardiograms on admission and during hospitalisation.**

(A) ECG on admission showing sinus tachycardia (114 bpm), incomplete right bundle branch block, right axis deviation, and nonspecific ST-T changes with T-wave flattening and inversion in multiple leads.

(B) ECG during hospitalisation showing sinus rhythm with persistent ST-T changes in leads I, II, and V3–V6.


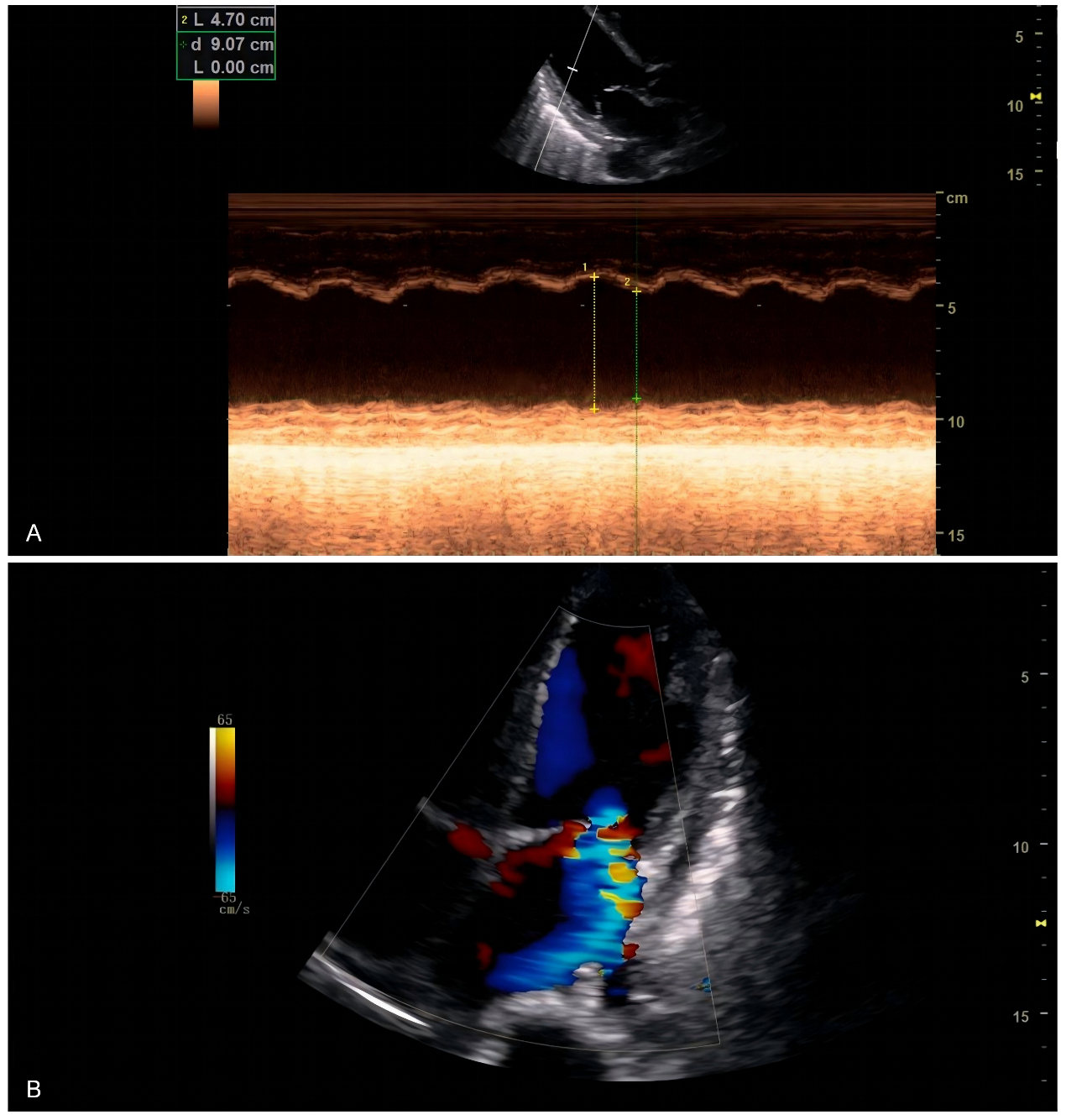


**Supplementary Figure S2. Transthoracic echocardiography at admission.**

(A) M-mode echocardiography showing left ventricular dilation and diffuse hypokinesis of the left ventricular walls.

(B) Apical four-chamber view confirming left ventricular dilation and diffuse wall motion abnormalities.

Quantitative measurements obtained from the examination are as follows:

Parameter Value Interpretation

EDV 167 mL Elevated (normal <147 mL)

ESV 120 mL Elevated (normal <61 mL)

SV 47 mL Within normal range (30-80 mL)

EF 28% Severely reduced (normal >50%)

The left ventricular ejection fraction was calculated using the biplane Simpson's method (EF = SV/EDV × 100% = (167-120)/167 × 100% ≈ 28%), demonstrating severely reduced systolic function consistent with fulminant myocarditis.
